# Medication use during end-of-life care in a palliative care centre

**DOI:** 10.1007/s11096-015-0094-3

**Published:** 2015-04-09

**Authors:** Anniek D. Masman, Monique van Dijk, Dick Tibboel, Frans P. M. Baar, Ron A. A. Mathôt

**Affiliations:** 1 Pain Expertise Centre, Erasmus MC-Sophia Children’s Hospital, Rotterdam, The Netherlands; 2 Palliative Care Centre, Laurens Cadenza, Rotterdam, The Netherlands; 3 Intensive Care, Department of Pediatric Surgery, Erasmus MC-Sophia Children’s Hospital, Room SK 1276, Dr. Molewaterplein 60, 3015 GJ Rotterdam, The Netherlands; 4 Hospital Pharmacy - Clinical Pharmacology, Academic Medical Centre, Amsterdam, The Netherlands

**Keywords:** Drug utilization, Drug therapy, Netherlands, Palliative care, Pharmaceutical preparations, Prescriptions, Terminal care

## Abstract

*Background* In end-of-life care, symptoms of discomfort are mainly managed by drug therapy, the guidelines for which are mainly based on expert opinions. A few papers have inventoried drug prescriptions in palliative care settings, but none has reported the frequency of use in combination with doses and route of administration. *Objective* To describe doses and routes of administration of the most frequently used drugs at admission and at day of death. *Setting* Palliative care centre in the Netherlands. *Method* In this retrospective cohort study, prescription data of deceased patients were extracted from the electronic medical records. *Main outcome measure* Doses, frequency and route of administration of prescribed drugs *Results* All regular medication prescriptions of 208 patients, 89 % of whom had advanced cancer, were reviewed. The three most prescribed drugs were morphine, midazolam and haloperidol, to 21, 11 and 23 % of patients at admission, respectively. At the day of death these percentages had increased to 87, 58 and 50 %, respectively. Doses of these three drugs at the day of death were statistically significantly higher than at admission. The oral route of administration was used in 89 % of patients at admission versus subcutaneous in 94 % at the day of death. *Conclusions* Nearing the end of life, patients in this palliative care centre receive discomfort-relieving drugs mainly via the subcutaneous route. However, most of these drugs are unlicensed for this specific application and guidelines are based on low level of evidence. Thus, there is every reason for more clinical research on drug use in palliative care.

**Electronic supplementary material:**

The online version of this article (doi:10.1007/s11096-015-0094-3) contains supplementary material, which is available to authorized users.

## Impact of findings on practice

- Nearing the end of life, patients in this palliative care centre receive discomfort relieving drugs mainly via the subcutaneous route. Most of these drugs are unlicensed, however, and optimal doses are unknown.
- Current palliative guidelines are mainly based on experience; prospective clinical trials are needed to formulate evidence base guidelines than can guide the choice and dose of drugs.
- Symptom assessment with validated instruments would be useful to taper drugs to the patients’ needs.

## Introduction

In 2011 approximately 136,000 persons died in the Netherlands, almost one-third of them from the consequences of cancer [1]. A systematic review on symptom prevalence in patients with incurable cancer found that the most reported symptoms were: fatigue (88 %), appetite loss (56 %), pain (45 %), dyspnea (39 %), drowsiness (38 %), dry mouth (34 %), constipation (29 %), confusion (24 %), nausea (17 %), and insomnia (14 %) [2].

The goal of palliative care is symptom control by a combination of non-pharmacological measures and drugs. Palliative experts have reached consensus on the essential drugs to treat specific symptoms. These have been compiled in two different but largely overlapping lists: one published by the International Association for Hospice and Palliative Care (IAHPC) [3] and one based on a survey of Australian palliative care physicians [4]. Regrettably, both lack recommendations on optimal dose or route of administration.

Existing recommendations [5, 6] on dose and route of administration are mainly based on level 3 and 4 evidence from case studies or from expert panels. Level 1 evidence from a systematic review or randomized controlled trials is available only for NSAIDs administered to relieve nociceptive pain [7] and morphine to alleviate dyspnea [8]. Level 3 evidence is available for the treatment of cancer pain with oral morphine [9]. Haloperidol treatment of a delirium in hospitalised patients is based on level 2 evidence from well designed, non-randomized trials [10]. Recent updates of systematic reviews for morphine and haloperidol found no new significant information [11, 12].

The choice of drug and dose tailored to the individual patient is thus hardly supported by evidence from prospective clinical trials. Likewise, there is little evidence for the optimal route of administration, although the subcutaneous route is often preferred in palliative care. Dose adjustment may be needed because liver and kidney function undergo changes at the end of life [13, 14]. It follows that a number of drugs used in palliative care are unlicensed or off-label [15, 16].

Only a few studies in palliative care units [17–19] and services for mainly outpatients groups [20–23] have described medication use in palliative care. To our knowledge, there are no published studies describing the most used drugs with their doses and administration routes, on admission and at the day of death in a large group of patients receiving palliative care.

## Aim of the study

The aim of this study was to evaluate what drugs were administered, and at what dose and route of administration, from admission to day of death in patients admitted to a single palliative care centre.

## Ethical approval

Ethical approval from a review board was not required, since this is a descriptive retrospective study. For retrospective analysis of patient files ethical approval is waived according to Dutch law. All patient data were handled and processed in accordance with the recommendations of Good Clinical Practice.

## Methods

### Design and setting

This retrospective cohort study was performed in Laurens Cadenza in Rotterdam, the Netherlands. This is the largest palliative care centre in the Netherlands, with 20 beds for terminal care and symptom management; from 200 to 250 patients are admitted annually. A multidisciplinary team of health care professionals is available 24 h per day.

### Measurements and technical information

Age, gender, primary diagnosis, comorbidities, and duration of admission were extracted from the electronic medical records. The primary diagnosis was assigned according to the WHO’s International Classification of Diseases (ICD-10 classification) coding for the patient’s terminal illness.

Medication data of all deceased patients in 2010 were extracted: name, dose, frequency, and route of administration, and dates of start and discontinuation of the prescription. Only the regular prescriptions for maintenance therapy were included, because the electronic prescription system does not detail how much as needed medication was given.

Drugs were prescribed according to the symptom-specific Dutch national palliative guidelines [5]. The presence of symptoms was daily checked by the nurses and reported to the physicians, but validated assessment instruments were not standard of care.

Two top-10 s of individual drugs prescribed were constructed: One covering the day of admission (Ta), the other the day of death (Td).

Medication was categorized by the anatomical therapeutic chemical (ATC) classification system [24]. The ATC system groups the drugs into 5 different levels according to the organ or system on which they act and according to their chemical, pharmacological and therapeutic properties. For this study we used the main therapeutic-group level. Furthermore, the WHO classification of analgesic drugs was applied: non-opioids, NSAIDs and opioids.

Morphine and haloperidol doses per 24 h were calculated taking into account route of administration. Oral bioavailability of morphine and haloperidol is 30 and 50 %, respectively, versus almost 100 % after subcutaneous, intravenous and intramuscular administration. Equivalent subcutaneous doses of oral drugs were calculated by dividing oral morphine doses by 3 and oral haloperidol doses by 2 [5, 25, 26]. An oral morphine dose of <300 mg/24 h is considered a low-to-moderate dose [27–29]. Consequently, a daily subcutaneous morphine dose of <100 mg/24 h was considered a low-to-moderate dose.

Fentanyl is mainly given via transdermal patches, which are replaced every 2–3 days. The daily dose was calculated as the dose of the prescribed patch divided by the number of days the patch was in place. Midazolam for continuous palliative sedation was administered either by subcutaneous boluses six times every 24 h or by constant subcutaneous infusion. Insomnia was mainly treated by a single subcutaneous bolus of midazolam or by intermittent boluses.

### Statistics

Data were analysed using descriptive statistics. Data are presented as mean (standard deviation; SD) in case of normally distributed variables and as median (interquartile range = IQR or minimum–maximum range = range) in case of non-normally distributed variables. IBM SPSS Statistics 20 was used for data analysis.

McNemar test served to detect differences in numbers of patients receiving the 3 most frequently used drugs both at Ta and Td. We limited ourselves to these three drugs to prevent repeated testing with too small samples. Differences in the daily doses of these drugs for patients receiving these both at Ta and Td were evaluated with the Wilcoxon signed rank test. A *p* value of <0.05 (two-sided) was deemed statistically significant.

## Results

### Participants

In the study year 2010, 234 patients had been admitted. Ten had been discharged in the course of 2010 and 16 were still alive at 1st January 2011. All other 208 patients died in the palliative care centre and were included for analysis. Their median age was 76 years (IQR 63–83 years), 50.5 % were female, and the median duration of admission was 11 days (IQR 5–29 days). Advanced malignancy, mainly of the digestive or respiratory organs, was the main reason for admission (88.9 % of patients). A median of two comorbidities (IQR 1–4) had been documented. Patient characteristics are given in Table 1.

**Table 1:** Background characteristics of the included patients

Characteristics	N = 208
Gender, in number (%)	
Male/female	103 (49.5)/105 (50.5)
Age, in years	
Median (IQR)	76 (63–83)
Duration of admission, in days	
Median (IQR)	11 (5–29)
Primary diagnosis, in number (%)	
Neoplasm	185 (88.9)
Digestive organs	50 (27.0)
Respiratory and intra-thoracic organs	47 (25.4)
Breast	13 (7.0)
Urinary tract	12 (6.5)
Unspecified or unknown sites	12 (6.5)
Lymphoid, hematopoietic and related tissue	10 (5.4)
Eye, brain and other parts of central nervous system	9 (4.9)
Male genital organs	8 (4.3)
Other	24 (13.0)
Disease of circulatory system	11 (5.3)
Other	12 (5.8)
Co-morbid conditions, in number	
Median (IQR)	2 (1–4)

### Prescriptions

Drug prescriptions had not been issued for two patients; one died quickly after admission and stayed for a few hours only, all medications for the other patient had already been discontinued shortly before admission. A total of 4890 prescriptions for 206 patients has been extracted, of which 3032 were regular prescriptions (62.0 %) for 203 patients. Regular prescriptions were issued for 194/198 (98.0 %) patients at Ta and for 202/206 (98.0 %) patients at Td.

The median number of drugs per patient at Ta was six (IQR 3–8) and this number had decreased to four (IQR 3–5) at Td.

### Top-10 individual regular drugs

**Fig. 1:**
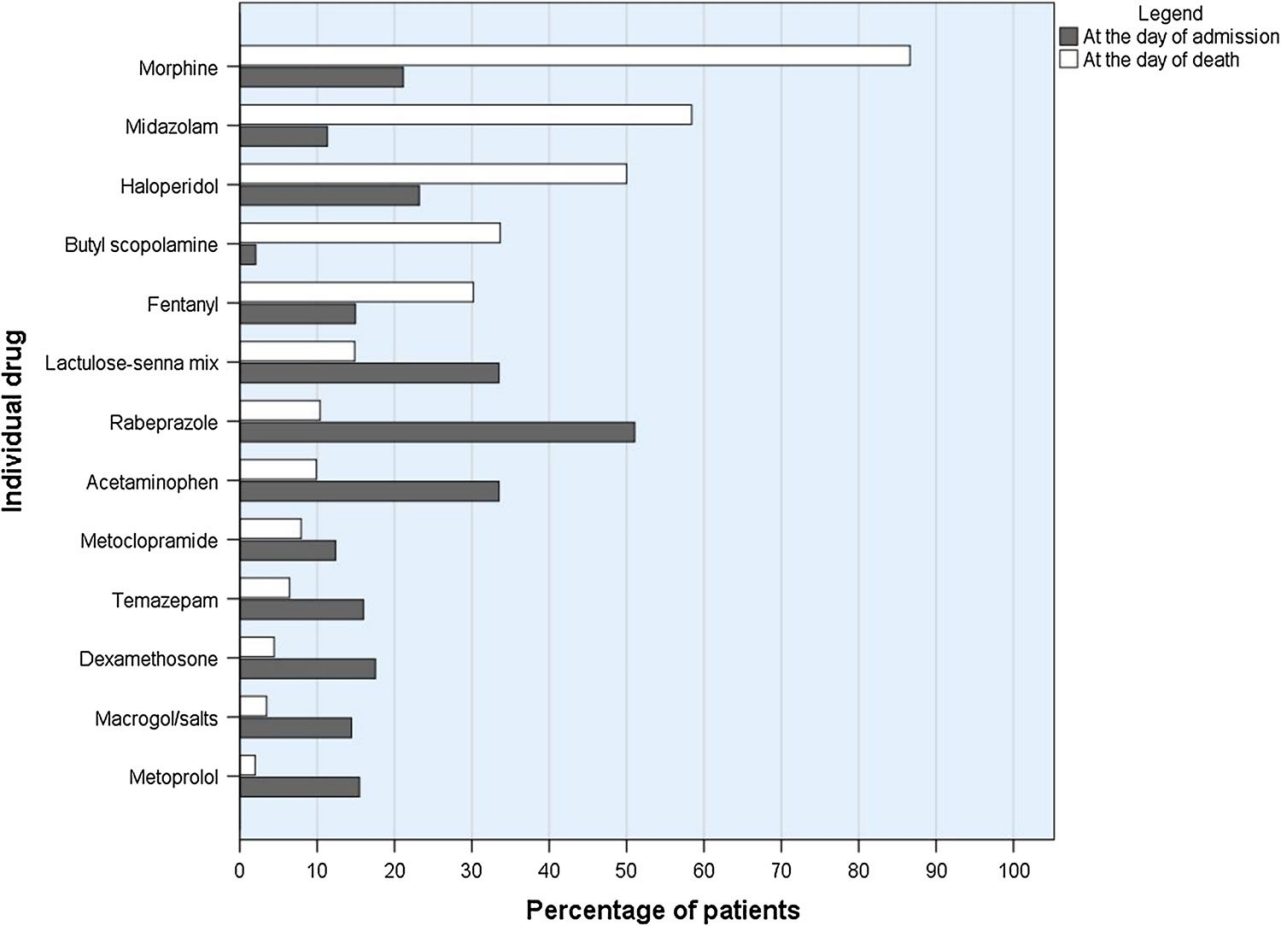
Differences in top-10 individual drugs at admission (*dark grey bars*) and at day of death (*white bars*); shown in descending order for the day of death

**Fig. 2:**
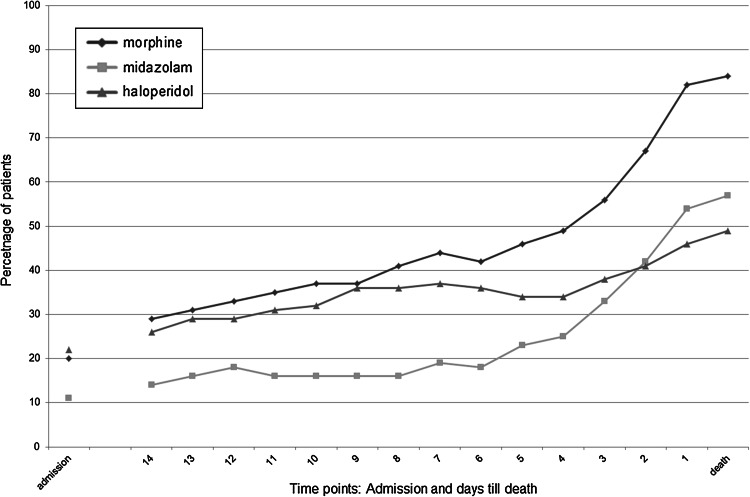
Prescriptions of top-3 drugs; morphine (*diamond*), midazolam (*square*) and/or haloperidol (*triangle*), at several time points during admission

The top-10 individual drugs prescribed at Ta and Td are given in Table 2. Figure 1 shows percentages of patients with a prescription of these top-10 drugs at Ta and Td. Morphine, midazolam, haloperidol, butyl scopolamine and fentanyl were prescribed more frequently at Td than at Ta. Numbers of patients with a prescription of morphine, midazolam or haloperidol increased statistically significantly from Ta to Td (all *p* values <0.001). This increase was most notable during the last week before Td as shown in Fig. 2. Prescriptions of lactoluse-senna mix, rabeprazole, acetaminophen, metoclopramide, temazepam, dexamethasone, macrogol/salts and metoprolol had been discontinued before Td.

**Table 2:** Top-10 individual regular drugs (in bold) at the day of admission (Ta) and the day of death (Td); given in descending order for the day of death

Individual drug top 10	Ta (N = 194)		Td (N = 202)	
	N (%)	Dose/24 h (median; IQR)	N (%)	Dose/24 h (median; IQR)
Morphine*	**41 (21.1)**	30 (17.5–60) mg	**175 (86.6)**	60 (30–65) mg
Midazolam	22 (11.3)	10 (5–10) mg	**118 (58.4)**	60 (20–90) mg
Haloperidol*	**45 (23.2)**	2 (range 0.25–4) mg	**101 (50.0)**	2 (range 0.5–5) mg
Butyl scopolamine	4 (2.1)	80 mg	**68 (33.7)**	80 (range 40–80) mg
Fentanyl	**29 (14.9)**	16.7 (8.3–25) mcg/hr	**61 (30.2)**	16.7 (8.3–25) mcg/hr
Lactulose-senna mix	**65 (33.5)**	15 (range 10–60) ml	**30 (14.9)**	15 (range 7.5–60) ml
Rabeprazole	**99 (51.0)**	20 (range 10–80) mg	**21 (10.4)**	20 (range 20–40) mg
Acetaminophen	**65 (33.5)**	4000 (range 1000–4000) mg	**20 (9.9)**	4000 (range 3000–4000) mg
Metoclopramide	24 (12.4)	40 (30–40) mg	**16 (7.9)**	40 (30–40) mg
Temazepam	**31 (16.0)**	10 (10–20) mg	**13 (6.4)**	10 (10–20) mg
Dexamethasone	**34 (17.5)**	8 (4–12) mg	9 (4.5)	8 (5.5–16) mg
Macrogol/salts	**28 (14.4)**	1 (1–2) sachets	7 (3.5)	1 (1–2) sachets
Metoprolol	**30 (15.5)**	50 (50–100) mg	4 (2.0)	50 (31.25–87.5) mg

* The route of administration is taken into account, the subcutaneous dose equivalent is given

Morphine, midazolam and haloperidol were often prescribed concomitantly (Table 3). Thirty-one per cent of the patients received all three at Td, but 11 % had neither a prescription of morphine, midazolam nor haloperidol at Td.

**Table 3:** Combination of prescription for top-3 drugs; morphine, midazolam and haloperidol (N = 202)

Single or combination of regular prescriptions at day of death	N (%)
Morphine, midazolam and haloperidol	63 (31.2)
Morphine and midazolam	46 (22.8)
Morphine	36 (17.8)
Morphine and haloperidol	30 (14.9)
No morphine, midazolam or haloperidol	22 (10.9)
Midazolam and haloperidol	6 (3.0)
Midazolam	3 (1.5)
Haloperidol	2 (1.0)

### Top-10 regular drug classes

Top-10 s of ATC drug classes prescribed at Ta and Td are given in supplementary Table S1. Three classes were prescribed more frequently at Td than at Ta: analgesics, psycholeptics and drugs for functional gastrointestinal disorders. While the top-10 at Ta included beta blocking agents, psycho-analeptics and anti-thrombotic agents, those drug classes were not included in the top-10 at Td. (Table S1, see supplement). Percentages of patients with a prescription of the top-10 drug classes at Ta and Td are shown in supplementary Figure S1.

Numbers of patients with analgesics classified by the different grouping systems are given in supplementary Table S2. The two most frequently prescribed opioids, i.e. morphine and fentanyl, are included in the top-10 of individual drugs in Table 2. The frequencies of combinations of prescriptions of non-opioids, NSAIDs and opioids are given in supplementary Table S3.

### Drug doses

The median daily doses for each individual drug prescribed at Ta and Td are displayed in Table 2. The median daily doses of the top-3 drugs at Td were: morphine 60 mg, midazolam 60 mg, and haloperidol 2 mg. Patients receiving these drugs both at Ta end Td were prescribed statistically significantly higher doses at Td than at Ta [morphine (n = 40) *p* < 0.001, midazolam (n = 18) *p* = 0.003 and haloperidol (n = 37) *p* = 0.028].

At Td, 83 % of the patients receiving morphine had a low-to-moderate subcutaneous equivalent morphine dose of <100 mg/24 h.

### Routes of administration

The three most common routes of administration were: oral (solid and liquids), subcutaneous, and transdermal. Percentages of patients with prescriptions of solid oral drugs declined from 89.2 % (n = 173) at Ta to 21.3 % (n = 43) at Td. Use of the subcutaneous route increased from Ta (47.9 %; n = 93) to Td (93.6 %; n = 189). Prescriptions of a transdermal drug almost doubled from Ta to Td, from 16.0 % (n = 31) to 31.7 % (n = 64) of patients (Table 4).

**Table 4:** Prescriptions via the various routes of administration at the day of admission (Ta) and the day of death (Td); given in descending order for the day of death

Routes of administration	Ta (N = 194)		Td (N = 202)	
	N (%)	Number of drugs per patient (median; IQR)	N (%)	Number of drugs per patient (median; IQR)
Subcutaneous	93 (47.9)	1 (1–2)	189 (93.6)	3 (2–3)
Transdermal	31 (16.0)	1	64 (31.7)	1
Oral, liquid	115 (59.3)	1	47 (23.3)	1
Oral, solid	173 (89.2)	4 (3–6)	43 (21.3)	3 (2–5)
Intramuscular	5 (2.6)		28 (13.9)	
Cutaneous*	15 (7.7)		16 (7.9)	
Inhalation	29 (14.9)		12 (5.9)	
Rectal	20 (10.3)		11 (5.4)	
Intravenous	4 (2.1)		3 (1.5)	
Ocular	4 (2.1)		2 (1.0)	
Intravesical	–		2 (1.0)	
Intrathecal	–		1 (0.5)	
Nasal	1 (0.5)		1 (0.5)	

* The cutaneous route is used for local skin treatment

Morphine, midazolam and haloperidol were almost exclusively given via the subcutaneous route. At Ta morphine was given subcutaneously to 95.1 % (39/41) of the patients, midazolam to 90.0 % (20/22) and haloperidol to 66.7 % (30/45). At Td these percentages had even increased to 98.9 % (173/175), 100 % (118) and 99 % (100/101) respectively.

## Discussion

This study found that morphine, midazolam and haloperidol were the most frequently prescribed drugs at the day of death for patients in the largest palliative care centre in the Netherlands. Doses of these drugs were statistically significantly higher than those at the day of admission. Upon admission almost 90 % of patients received oral medication but over the admission period a shift occurred to the effect that at the day of death more than 90 % of patients received subcutaneous medication.

Other studies, too, found that morphine, midazolam and haloperidol were the most prescribed drugs in the palliative setting [30–33]. These drugs are given to relieve symptoms such as pain, restlessness and agitation, which are frequently seen in advanced cancer [2]. Nauck and co-workers [17] in a similar study found that 26 % of patients received morphine at admission (versus 21 % in the present study), but corresponding figures at the end of treatment were 42 versus 87 %. The latter difference is probably explained by the fact that Nauck and co-workers also included patients who were discharged from the centre, whereas we solely considered patients who died in the palliative centre. Nevertheless, other studies reported opioid use in 82–97 % [28, 30, 32], and morphine use in 66–93 % [27, 28, 30, 32] of patients at the end of life, which percentages correspond well with our results.

We found that midazolam was prescribed for 58 % of patients at the day of death, while in other studies this was the case for 23 % of patients in the last 48 h of life [30] or 82 % of patients in their last week [31]. An explanation for this wide range could be the studied time frame. Midazolam is often stopped in the last days before death, to avoid that patients become comatose. On the other hand, midazolam may be started for palliative sedation, notably in the last 24 h before death.

Many more patients in the present study were prescribed haloperidol than in the study by Nauck et al. [17]; at admission 23 versus 3 %, respectively, and at end of treatment 50 versus 13 %, respectively. Our higher figures may be explained by the difference in the studied patient population; we only included patients who died in the palliative centre. Other studies, however, found percentages (21–43 %) comparable to the present study [30–33]. Haloperidol is the drug of first choice to treat delirium. In other studies, delirium was suspected in approximately 50 % of cancer patients admitted to a palliative care centre and in up to almost 90 % of all cancer patients in the last day or hours before death [34, 35]. We suspect, however, that haloperidol is also prescribed in agitated or restless patients who have not been clearly diagnosed with a delirium. Therefore, assessing delirium with a validated scale, such as the Confusion Assessment Method, should become standard of care. [36, 37].

In the present study the median number of drugs decreased from 6 to 4 as death approached, probably because in our centre oral drugs are stopped when a patient enters a recognizable dying phase [38]. Other studies, however, have reported increasing numbers of drugs towards death [20, 22, 23], possibly to control a new or advancing symptom.

The doses of the top-10 drugs compared well to the titration schemes given in the national symptom specific guidelines [5]. Eighty-three percent of patients in the present study received a subcutaneous morphine dose of <100 mg/24 h at the day of death, which is considered a low-to-moderate dose [27–29]. In two other studies more than 90 % of the patients received low-to-moderate morphine doses either upon admission [27] or in the last 24 h before death [28] .

The median subcutaneous midazolam dose (60 mg/24 h) at the day of death in the present study fits within the range found in other studies; mean midazolam doses of 26–70 mg/24 h during the last days of life [30, 31, 39]. Moreover, these doses (IQR 30-65 mg/24 h, in present study) are recommended in the Dutch national guideline for palliative sedation [5]. However, midazolam dose titration should be guided by regular assessment of level of sedation.

The median haloperidol dose was 2 mg/day, both at admission and the day of death. Other studies found median haloperidol doses of 2.5–3.8 mg/day during the last days of life [30, 32]. The Dutch national guideline for delirium treatment, however, recommends a maximum parenteral maintenance dose of 10 mg/day [5]. In practice the recommended starting dose of 0.5–2 mg/day seems sufficient to treat delirium in most patients. Moreover, in elderly patients a low starting dose is recommended to prevent neurological and cardiovascular effects [25].

Over the admission period a shift occurred from the oral route to mainly the subcutaneous route, in line with recommendations from both the guidelines [5, 6] and the Liverpool Care Pathway for the dying [38]. The subcutaneous route is preferred in palliative care because most patients are unable to take oral medication at the end of life and the intravenous route is often complicated by infection or discomfort. Absorption via the subcutaneous route may be suboptimal, however, especially in cachectic cancer patients with very little or no subcutaneous fat.

Although the subcutaneous route is preferred in palliative care, this route has not been fully studied. In addition, midazolam and haloperidol are unlicensed or off-label in this patient group [15, 16, 40, 41]. Regarding opioids, only small and mostly non-randomized controlled clinical trials have compared the subcutaneous route with another route of administration [12, 42]. In those studies similar feasibility, efficacy and opioid doses were found for the subcutaneous route and the intravenous route. Moreover, in some studies the subcutaneous route was preferred because of lower complication risks. Only small and outdated prospective studies are available for midazolam, which all found subcutaneous administration of midazolam to be feasible and effective [39, 43, 44]. Regarding haloperidol, only retrospective descriptive studies or overview articles are available, even without addressing the administration route [45–48]. In conclusion, strict monitoring of the efficacy of subcutaneous morphine, midazolam and haloperidol is essential and more pharmacokinetic and pharmacodynamics studies are needed.

### Strengths and limitations

A strength of the present study is that actually administered regular medication in the palliative care setting was evaluated at two significant time points, detailing drug doses and routes of administration. In addition, electronically recorded prescriptions were available, preventing the errors of written medication orders when extracting data.

Several limitations should be addressed however. First, this was a single-centre study of which the results cannot be extrapolated to other palliative care settings or other countries as prescription practices may differ. Second, as needed prescriptions were excluded from analysis, since our electronic prescription system did not detail how much as needed medication was actually given. In our centre, ‘as needed’ prescriptions mainly serve to increase the already prescribed doses of the medications, for example when worsening of symptoms is expected. When a patient is given the ‘as needed’ medication on a regular basis, the maintenance prescription dose is adapted accordingly. Unfortunately, also indications for drugs could not be analysed, since this information was not electronically recorded. In future research, both the as needed medication and the indications should be included, so as to provide a complete overview of administered symptom-specific drugs. Lastly, outcomes of validated assessment instruments for pain, sedation and delirium were not available. In future research these assessments should be included to add information on the efficacy of drugs.

### Recommendation

From the above it follows that pharmacotherapy in palliative care offers room for improvement. Therefore, we would recommend to strictly monitor the efficacy of the subcutaneously administered drugs with the use of validated pain, sedation and delirium assessment instruments. This will help recognize worsening of symptoms and enable to taper treatment to a patient’s needs.

## Electronic supplementary material

Below is the link to the electronic supplementary material.

Supplementary material Table S1 (DOCX 26 kb)

Supplementary material Table S2 (DOCX 26 kb)

Supplementary material Table S3 (DOCX 20 kb)

Supplementary material Figure S1 (TIFF 283 kb)
