# Supplementary material for: Medication use during end-of-life care in a palliative care centre
Source: Int J Clin Pharm. 2015 Apr 9;37(5):767–75. doi: 10.1007/s11096-015-0094-3 (PMC4594093; doi:10.1007/s11096-015-0094-3)
Supplement: Supplementary file 1 — Supplementary material Table S1 (DOCX 26 kb) [file 11096_2015_94_MOESM1_ESM.docx]

Supplement TableS1. Top-10 ATC drugs classes (in bold) at admission (Ta) and the day of death (Td); given in descending order for the day of death

| **Drug classes**  **Top 10** | **Ta (N=194)** | **Td (N=202)** |
| --- | --- | --- |
|  | **N (%)** | **N (%)** |
| Analgesics | **110 (56.7)** | **191 (94.6)** |
| Psycholeptics | **103 (53.1)** | **161 (79.7)** |
| Drugs for functional gastrointestinal disorders | **36 (18.6)** | **84 (41.6)** |
| Laxatives | **100 (51.5)** | **37 (18.3)** |
| Drugs for acid related disorders | **118 (60.8)** | **28 (13.9)** |
| Corticosteroids for systemic use | **55 (28.4)** | **16 (7.9)** |
| Anti-epileptics | **31 (16.0)** | **14 (6.9)** |
| Dermatological preparations | 12 (6.2) | **13 (6.4)** |
| Drugs for obstructive airway disease | 29 (14.9) | **12 (5.9)** |
| Diuretics | 28 (14.4) | **12 (5.9)** |
| Beta blocking agents | **40 (20.6)** | 8 (4.0) |
| Psycho-analeptics | **31 (16.0)** | 8 (4.0) |
| Antithrombotic agents | **52 (26.8)** | 5 (2.5) |
