# Supplementary material for: Medication use during end-of-life care in a palliative care centre
Source: Int J Clin Pharm. 2015 Apr 9;37(5):767–75. doi: 10.1007/s11096-015-0094-3 (PMC4594093; doi:10.1007/s11096-015-0094-3)
Supplement: Supplementary file 2 — Supplementary material Table S2 (DOCX 26 kb) [file 11096_2015_94_MOESM2_ESM.docx]

Supplement TableS2. Analgesics at admission (Ta; N=194) and at the day of death (Td; N=202); given in descending order for the individual drugs per drug class at the day of death

| **ATC therapeutic subgroup** | **WHO groups** | **Ta** | **Td** | **Individual drugs** | **Ta** | **Td** |
| --- | --- | --- | --- | --- | --- | --- |
|  |  | **N (%)** | **N (%)** |  | **N (%)** | **N (%)** |
| Analgesics | Opioids | 82 (42.3) | 187 (92.6) | Morphine | 41 (21.1) | 175 (86.6) |
|  |  |  |  | Fentanyl | 29 (14.9) | 61 (30.2) |
|  |  |  |  | Oxycodone | 16 (8.2) | 4 (2.0) |
|  |  |  |  | Hydromorphone | 1 (0.5) | 2 (1.0) |
|  |  |  |  | Tramadol | 5 (2.6) | 1 (0.5) |
|  | Non-opioids | 66 (34.0) | 20 (9.9) | Acetaminophen  +codeine | 65 (33.5)  1 (0.5) | 20 (9.9)  - |
| Anti-inflammatory and anti-rheumatic drugs | NSAIDs | 19 (9.8) | 5 (2.5) | Diclofenac | 11 (5.7) | 3 (1.5) |
|  |  |  |  | Ibuprofen or naproxen | 4 (2.1) | 2 (1.0) |
|  |  |  |  | Celecoxib or etoricoxib | 4 (2.1) | - |
