# Supplementary material for: Medication use during end-of-life care in a palliative care centre
Source: Int J Clin Pharm. 2015 Apr 9;37(5):767–75. doi: 10.1007/s11096-015-0094-3 (PMC4594093; doi:10.1007/s11096-015-0094-3)
Supplement: Supplementary file 3 — Supplementary material Table S3 (DOCX 20 kb) [file 11096_2015_94_MOESM3_ESM.docx]

Supplement TableS3. Combinations of analgesics according to the WHO grouping at admission (Ta) and the day of death (Td); given in descending order for the day of death

| **Single or combination of regular analgesics** | **Ta (N=194)** | **Td (N=202)** |
| --- | --- | --- |
|  | **N (%)** | **N (%)** |
| Single opioid | 36 (18.6) | 119 (58.9) |
| Combination of opioids | 3 (1.5) | 49 (24.3) |
| Non-opioid and opioid(s) | 33 (17.0) | 15 (7.4) |
| Single non-opioid | 24 (12.4) | 3 (1.5) |
| NSAID and opioid(s) | 5 (2.6) | 3 (1.5) |
| Non-opioid, NSAID and opioid(s) | 5 (2.6) | 1 (0.5) |
| Non-opioid and NSAID | 4 (2.1) | 1 (0.5) |
| Single NSAID | 5 (2.6) | - |
