# Supplementary figures and images for: Medication use during end-of-life care in a palliative care centre
Source: Int J Clin Pharm. 2015 Apr 9;37(5):767–75. doi: 10.1007/s11096-015-0094-3 (PMC4594093; doi:10.1007/s11096-015-0094-3)

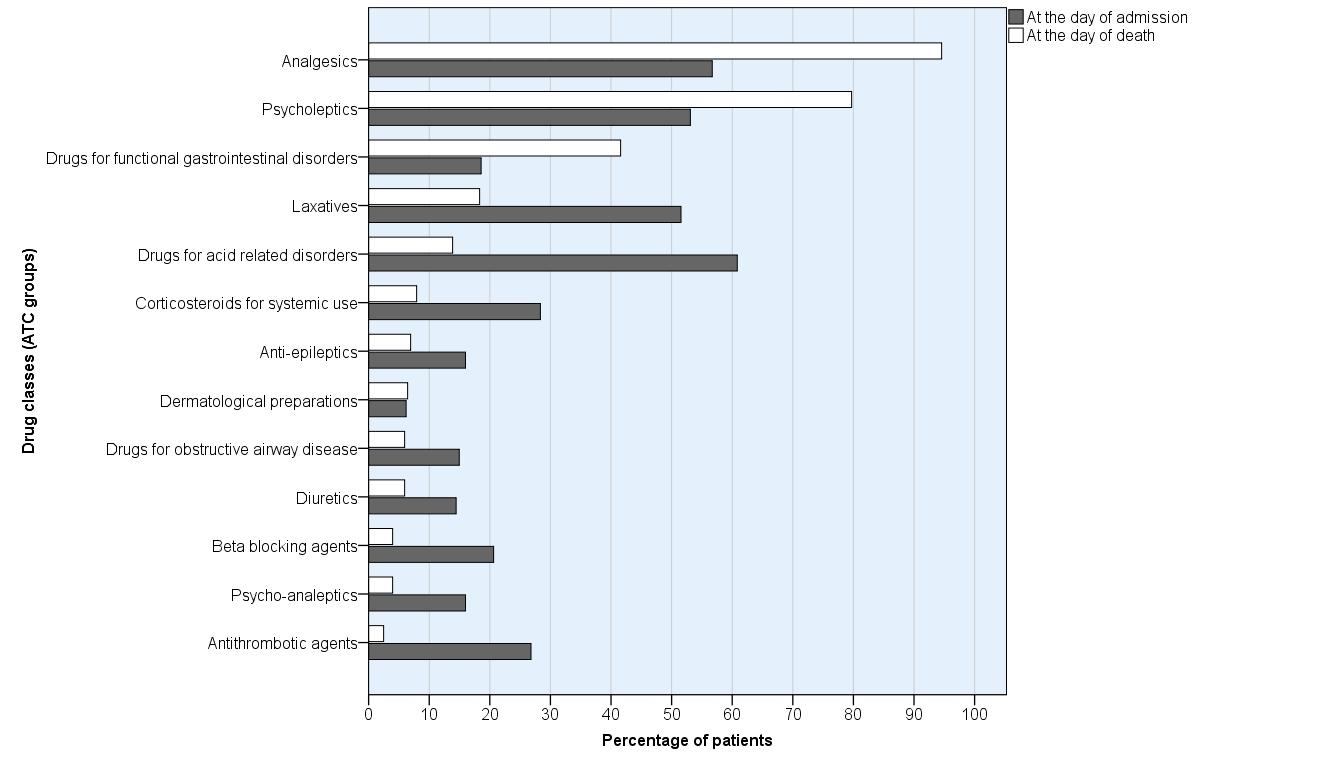

Supplement: Supplementary file 4 — Supplementary material Figure S1 (TIFF 283 kb) [file 11096_2015_94_MOESM4_ESM.tif]
